# Health Care Systems and Conflict: A Fragile State of Affairs

**DOI:** 10.1371/journal.pmed.1001065

**Published:** 2011-07-26

**Authors:** The PLoS Medicine Editors

## Abstract

In this month's editorial, the *PLoS Medicine* Editors discuss the impact of armed conflict on health care systems and announce their support of the International Health Protection Initiative's resolution to reinforce the Geneva Conventions.

The current debates in the US over the constitutionality of the 2010 Patient Protection and Affordable Care Act's mandate that individuals purchase health insurance, and in England about the government's plans to radically reform the National Health Service, emphasize the importance of health systems and of the quality of care they provide to entire nations. While health care systems are necessary in all countries, the importance of strong health care systems to fragile nations, and the damage done to these systems during conflict, receive less attention than they should.

There are various definitions of what constitutes a fragile country or society [1], but most agree that a fragile state is one in which the government is unable or unwilling to deliver basic security and public services to the majority of its people, especially to the poor. These countries are frequently torn by armed conflict and plagued by high rates of poverty, creating a vicious cycle from which it is difficult to emerge. A recent assessment put the number of fragile and conflict-affected states at about 30 [2], although this may change in light of recent events such as the secession of Southern Sudan and the ongoing volatile situations in North Africa and the Middle East.

The impact of this cycle of violence and poverty on health and health care is enormous. According to the World Bank's World Development Report 2011 [3], no low-income fragile or conflict-affected country has yet achieved a single Millennium Development Goal. People living in fragile states are more than twice as likely as those in stable developing countries to be undernourished and to lack clean water; the mortality rate for children under the age of five is twice as high. And although the international community spends billions of dollars each year in aid to these nations, gains have generally been small; without infrastructure and stability, much of this aid is wasted.

But there are health-related success stories. Afghanistan's history is typical of a fragile nation: in 2003, after decades of conflict, the country had terrible poverty rates, an infant mortality rate estimated at 165 per 1,000 live births, and an under-five mortality rate estimated to be 257 per 1,000 live births – some of the highest rates in the world [4]. In 2004, a Basic Package of Health Services was introduced by Afghanistan's Ministry of Public Health, and a balanced scorecard system was adopted to measure and manage the performance of health systems and services [5]. In this week's issue of the journal, Anbrasi Edward and colleagues report on the development and performance of Afghanistan's health care services between 2004 and 2008, demonstrating dramatic improvements in many areas, especially in health service capacity and delivery of care [6].

In spite of these impressive gains, the future of health care in Afghanistan, and indeed the likelihood of Afghanistan emerging from its fragile status, is far from certain. While significant progress has been made in reducing infant and under-five child mortality rates by 22% and 26%, respectively [4], over 35% of the population continues to live in poverty, a percentage which has remained virtually unchanged over the last 10 years [7]. The country has also been dependent on international donors to financially support its rebuilding and redevelopment; in the current economic climate, diminishing donor aid poses a significant hurdle to maintaining and increasing the gains made in the country to date.

And in some ways, the findings in this study are a best-case scenario of the current health situation in Afghanistan. Six of the 34 provinces in the country were not included in this analysis; five of them – Helmand, Kandahar, Zabul, Uruzgan, and Farah – were excluded because of worsening security situations. These security issues threaten not just the evaluation of health care provision, but the health care services and providers themselves – a complication virtually unknown in stable nations.

Within the last year medical personnel have been attacked and killed in Afghanistan, even in the more peaceful areas within the country [8]. Although banned by international humanitarian law, the targeting of health care infrastructure and personnel during armed conflict occurs with alarming regularity. Indeed, within the last few months, aid organizations and the international media have reported that hospitals, ambulances, and aid workers have been attacked during the current conflict in Libya [9]–[11], just one of many ongoing armed conflicts where health care systems and workers have been targeted.

Events such as these have led to the establishment of a new resolution by the International Health Protection Initiative to reinforce the Geneva Conventions and their associated protocols, a resolution to which this journal has pledged its support. This resolution in part calls on states to comply with international humanitarian law and to prosecute those responsible for attacks on health care facilities, health care workers, patients, and transport vehicles. Further, it calls on the World Health Organization to “provide guidance to member States in how to increase protection of health functions in zones of armed conflict” [12].

The development of fragile states is complex: security must be provided and maintained, unemployment addressed, and education fostered. The adequate and equitable provision of quality health care is also, however, a fundamental need. This need will be met only if health systems and structures are preserved and developed, and if health care personnel have the freedom and safety to provide necessary care to those who need it.
